# Rotational thromboelastometry predicts thromboembolic complications after major non-cardiac surgery

**DOI:** 10.1186/s13054-014-0549-2

**Published:** 2014-10-08

**Authors:** Alexander Hincker, Justin Feit, Robert N Sladen, Gebhard Wagener

**Affiliations:** Department of Anesthesiology, College of Physicians and Surgeons, Columbia University, P&S Box 46 (PH-5), 630 West 168th Street, New York, NY 10032-3784 USA

## Abstract

**Introduction:**

Thromboembolic complications contribute substantially to perioperative morbidity and mortality. Routine laboratory tests do not detect patients with acquired or congenital hypercoagulability who may be at increased risk of perioperative thromboembolism. Rotational thromboelastometry (ROTEM) is a digitized modification of conventional thromboelastography that is stable and technically easy to use. We designed a prospective observational study to evaluate whether preoperative ROTEM can identify patients at increased risk for postoperative thromboembolic complications after major non-cardiac surgery.

**Methods:**

Preoperative ROTEM analysis using extrinsic rotational thromboelastometry (EXTEM), intrinsic rotational thromboelastometry (INTEM), and fibrinogen rotational thromboelastometry (FIBTEM) activators was performed on 313 patients undergoing major non-cardiac surgery. Patients’ medical records were reviewed after discharge for results of standard coagulation studies - partial thromboplastin time (PTT), international normalized ratio (INR), platelet count - and evidence of thromboembolic complications during their hospital stay. A thromboembolic complication was defined as a new arterial or deep venous thrombosis, catheter thrombosis, or pulmonary embolism diagnosed by ultrasound or spiral chest computed tomography.

**Results:**

Ten patients developed postoperative thromboembolic complications, of whom 9 had received standard prophylaxis with subcutaneous enoxaparin or heparin. There was no indication of by PTT, INR, or platelet count. Preoperative EXTEM and INTEM activators that assess fibrin clot formation and platelet interaction indicated that these patients had significantly lower clot formation time (CFT) and significantly higher alpha angle (α) and maximum clot firmness (MCF), compared to patients without thromboembolic complications. There was no significant difference for any parameter using FIBTEM activator, which excludes platelet interaction. Receiver operating characteristic (ROC) curves were constructed for these variables. INTEM clot firmness at 10 min (A10) was the best predictor of thromboembolic complications, with an ROC area under the curve of 0.751.

**Conclusions:**

Our results indicate that preoperative ROTEM assays that include fibrin clot and platelet interaction may detect patients at increased risk for postoperative thromboembolic complications after major non-cardiac surgery. Future studies need to evaluate the clinical utility and cost effectiveness of preoperative ROTEM and better define the association between ROTEM values and specific hypercoagulable conditions.

## Introduction

Thromboembolic complications of surgery such as deep vein thrombosis and pulmonary embolism induce substantial postoperative morbidity and mortality [1]. Postoperative anticoagulation to prevent these complications may in its turn increase morbidity by inducing major bleeding [2]. Certain patient and operative characteristics are known to predispose patients to postoperative thromboembolic events [3], and complex scoring systems have been developed to detect patients at increased risk [4]. However, neither scoring systems nor routine coagulation tests detect patients with acquired or congenital hypercoagulable states. For example, up to 8% of the European population is heterozygous for Factor V Leiden [5]. These patients are at increased risk for thromboembolic complications, but are not easily identified prior to surgery. The development of a single, readily available test to identify patients at increased risk for postoperative venous thromboembolism could provide early identification, guide more effective thromboembolic prophylaxis, and result in improved perioperative outcomes.

Rotational thromboelastometry (ROTEM) is a modular, computerized point-of-care system that is based upon a modification of the principles of thromboelastography (TEG). ROTEM utilizes a variety of activators to provide a targeted and dynamic analysis of specific aspects of the coagulation cascade [5]. As such, ROTEM can provide a more detailed evaluation of clot formation and breakdown in the individual patient and can identify specific cascade abnormalities. The present study was designed to determine the ability of preoperative ROTEM analysis to predict postoperative thromboembolic complications in patients undergoing major non-cardiac surgery.

## Materials and methods

### Study design and subjects

All adult patients undergoing major non-cardiac surgery at Columbia University Medical Center were eligible for enrollment. We only used blood that was to be discarded during clinically indicated blood draws. The Columbia University Institutional Review Board (IRB) approved this study and waived the requirement to obtain written preoperative consent. In accordance with US Federal Guidelines, it considered the risk of this study minimal and agreed that the study could not practicably have been carried out without a waiver of consent. All patients or their surrogates received an information sheet after surgery to inform them about this study.

Major surgery was defined as any elective non-cardiac surgical procedure that was likely to require inpatient admission for more than three days, as well as the intraoperative placement of an arterial catheter for blood draws. Patients were the first cases each day in the operating rooms, randomly selected with no specific randomization scheme. History of past or current thromboembolism prior to surgery did not exclude patients from the study, but persistence of a known preoperative condition was not considered a positive postoperative thromboembolic event. A thromboembolic complication was defined as a new arterial or deep venous thrombosis, catheter thrombosis, or pulmonary embolism diagnosed by ultrasound or spiral computed tomography (CT) during hospitalization after the surgical procedure. A positive thromboembolic complication was determined by review of all charts for diagnostic tests that would be used to diagnose thromboembolic complications, such as ultrasound or spiral CT. Clinicians caring for the patients were not given access to the ROTEM results, and routine anticoagulation regimens were used as directed by the clinicians. No additional tests were ordered to evaluate for thromboembolic complications.

### Data and sample collection

Two milliliters of whole blood was collected for ROTEM analysis from preoperatively drawn blood that was left over from blood sent for routine scheduled laboratory tests and would otherwise have been discarded (waste blood). The blood was immediately filled into a citrated tube and ROTEM analysis was performed within 60 minutes. Of note, ROTEM results have been shown to be reproducible using blood samples stored up to 120 minutes at room temperature [6], and potentially longer. Medical records were reviewed for patient demographics, surgical procedure, preoperative laboratory test results including international normalized ratio (INR), partial thromboplastin time (PTT) and platelet count, ICU and hospital length of stay (LOS), and radiological and/or clinical evidence of a thromboembolic complication.

### ROTEM analysis

Coagulability was assessed using the ROTEM thromboelastometry analyzer (Tem Systems Inc.®, Munich, Germany). A detailed description of the ROTEM technology has been published previously [7]. Extrinsic rotational thromboelastometry (EXTEM), intrinsic rotational thromboelastometry (INTEM), and fibrinogen rotational thromboelastometry (FIBTEM) tests were displayed on each blood sample. Each ROTEM test requires approximately 300 microliters of citrated whole blood. All samples in this study were processed within less than one hour after collection, given that ROTEM results are unchanged for citrated samples stored at room temperature for up to 2 hours [6] and potentially as long as up to six hours [8]. Quality control tests were run every week using normal and abnormal control plasma samples with known output parameters.

### Statistical methods

ROTEM results were analyzed two ways. First, clotting time, clot formation time (CFT), alpha angle (α), clot firmness at 10 minutes (A10), and maximum clot firmness (MCF) for each of intrinsic rotational thromboelastometry (INTEM), extrinsic rotational thromboelastometry (EXTEM), and fibrinogen rotational thromboelastometry (FIBTEM) were compared between patients with and without postoperative thromboembolic complications, using Student’s *t*-test or the Mann-Whitney (Wilcoxon rank) test (using α <0.05). Comparisons and correlations between groups were made by the unpaired *t*-test or Pearson’s test for correlation between values with a Gaussian distribution and by the Mann-Whitney test or Spearman’s test for correlation between continuous variables without normal distribution. Gaussian distribution was determined using Levene’s test for unequal variance. Categorical data were compared using the Chi-square or Fisher exact test. *P*-values were two-tailed and *P* <0.05 was considered significant.

Receiver operating characteristic (ROC) curves were plotted [9] and the point on the ROC curve closest to sensitivity = specificity =1 was considered the best cutoff value. We additionally calculated the Youden index (sensitivity + specificity −1) and used the largest Youden index as another best cutoff value. The area under the curve (AUC) of the ROC curve was estimated using Mann-Whitney statistics with associated Wald 95% confidence intervals.

The MCF is the most commonly used parameter to detect hypercoagulability [10], and is analogous to maximum amplitude (MA) in the TEG [11,12]. We compared the MCF for each of EXTEM, INTEM, and FIBTEM to established reference values for the general population [8]. A test was defined as hypercoagulable if the patient’s MCF was greater than that of the 97.5^th^ percentile of the general population, that is, above the reference range. Reference values are depicted in Table 1, second column. There are no reliable reference values described for FIBTEM α and A10, therefore we excluded these variables. We defined hypercoagulability by an appropriate MCF with any single test, with any two out of three tests, or with EXTEM activator. We then used the Chi-square test (α <0.05) to examine whether patients who were preoperatively hypercoagulable by ROTEM had higher rates of postoperative thromboembolic complications than those who were not.

**Table 1:** **Preoperative rotational thromboelastometry (ROTEM) parameters**

**ROTEM parameter**	**Normal values**	**No thromboembolic complication (n =303), mean (SD)**	**Thromboembolic complication (n =10), mean (SD)**	***P*** **-value**	**AUC ROC**
**EXTEM**					
Clotting time, s	42 to 74	55.1 (31.2)	48.7 (16.9)	0.19	---
Clot formation time, s	46 to 148	87.1 (61.7)	58.5 (16.6)	<0.001	0.74
Alpha angle, degrees	63 to 81	74.3 (6.9)	78.4 (3.1)	0.002	0.70
Amplitude at 10/20 minutes, mm	50 to 69	58.2 (8.7)	64.0 (5.4)	0.008	0.72
Maximum clot firmness, mm	49 to 71	65.0 (7.5)	70.4 (5.2)	0.009	0.73
**INTEM**					
Clotting time, s	137 to 246	171.5 (34.1)	165.8 (24.6)	0.11	---
Clot formation time, s	71 to 82	72.2 (57.3)	51.0 (11.4)	<0.001	0.75
Alpha angle, degrees	52 to 72	76.5 (5.5)	79.4 (2.4)	0.006	0.72
Amplitude at 10/20 minutes, mm	137 to 246	56.7 (7.8)	63.0 (5.8)	0.012	0.75
Maximum clot firmness, mm	52 to 72	62.8 (7.1)	68.6 (6.0)	0.02	0.74
**FIBTEM**					
Clotting time, s	43 to 69	53.3 (47.4)	44.7 (13.1)	0.11	---
Clot formation time, s		339.9 (366.5)	378.9 (606.0)	0.12	---
Alpha angle, degrees		73.6 (6.6)	77.0 (5.4)	0.085	---
Amplitude at 10/20 minutes, mm	8 to 21	16.5 (6.8)	23.7 (11.2)	0.010	---
Maximum clot firmness, mm	9 to 25	17.8 (7.6)	24.8 (11.2)	0.015	---

For significant predictive tests, the area under the curve (AUC) of the receiver operating characteristic (ROC) curve is listed. EXTEM, extrinsic rotational thromboelastometry; INTEM, intrinsic rotational thromboelastometry; FIBTEM, fibrinogen rotational thromboelastometry; ns, not significant.

SPSS 11.0.4 (SPSS Inc., Chicago, IL, USA) and Graphpad Prism 4.0 (San Diego, CA, USA) software were used for the statistical analysis.

## Results

We enrolled and successfully performed ROTEM analysis on 318 patients undergoing major non-cardiac surgery between April and August 2012. Of these, five patients were excluded from the final analysis. Two patients chose not to participate in the study and one patient underwent an operation that did not constitute major surgery (uncomplicated shoulder arthroscopy). One patient’s hospital records were insufficient for determination of whether or not she had suffered any thromboembolic events, and one patient’s radiologic examination could not definitively determine whether the patient’s thromboembolic complication occurred before or after surgery.

Of the 313 included patients, 10 (3.2%) suffered thromboembolic complications prior to their discharge from the hospital. These included six patients with isolated deep vein thrombosis, three patients with deep vein thrombosis further complicated by pulmonary embolism, and one patient who developed both a common femoral artery aneurysm thrombosis and a thrombosis of a vascular catheter. One patient died as a direct result of his pulmonary embolism. The thromboembolic complications occurred between 1 and 17 days after surgery (median 5 days, interquartile range: 1.8 to 7.25 days). With the exception of one patient who developed a thromboembolic complication on postoperative day one, all received standard postoperative thromboembolic prophylaxis. Four patients received 40 mg enoxaparin subcutaneously once a day and five patients received 5000 IU heparin subcutaneously three times daily from the day of surgery up to the day when the thromboembolic complication was diagnosed.

Patient demographic information and standard coagulation studies (PTT, INR, platelet count) are summarized in Table 2. Standard coagulation studies represent the unweighted average of all such tests taken on postoperative day zero. Previously, standard coagulation studies have been correlated with ROTEM parameters, for example in patients undergoing major surgery with hemorrhage [13]. In patients who developed coagulopathy and bleeding, abnormal platelet counts, INR and fibrinogen levels correlated well with EXTEM and INTEM, MCF and other ROTEM values. However, the standard coagulation studies did not provide any indication of which patients might be hypercoagulable. In our investigation, standard coagulation studies, type of surgery, and patient demographic factors were not significantly different in patients with or without postoperative thromboembolic complications (*P* >0.05 for all). Patients who suffered thromboembolic complications had noticeably longer mean ICU LOS (5.7 +/−9.7 versus 0.9 +/−3.4 days) and hospital LOS (25.5 +/−32.4 versus 6.2 +/−7.8 days). However, these differences were not statistically significant when no Gaussian comparison was used (median (interquartile range) ICU LOS: 2.5 (2 to 4) versus 0 (0 to 1) days, *P* >0.05; hospital LOS: 11.5 (8.75 to 33.8) versus 5 (3 to 7) days, *P* >0.05).

**Table 2:** **Patient demographics, outcomes and standard coagulation studies**

	**All (n =313)**	**No thromboembolic complication (n =303)**	**Thromboembolic complication (n =10)**	***P*** **-value**
Age, years	58.7 (+/−15.2)	58.5 (+/−15.2)	63.6 (+/−16.1)	0.30
Body mass index, kg/m^2^	27.7 (+/−6.3)	27.8 (+/−6.3)	26.4 (+/−4.8)	0.49
Obesity	102 (32.6%)	99 (32.7%)	3 (30%)	0.57
Female	165 (53%)	161 (53%)	4 (40%)	0.88
**Surgery class**				
Major abdominal	85 (27%)	81 (27%)	4 (40%)	0.47
Gynecological	4 (1%)	4 (1%)	0 (0%)	1.0
Central nervous system neurosurgery	48 (15%)	48 (16%)	0 (0%	0.37
Spine	62 (20%)	60 (20%)	2 (20%)	1.0
Vascular	11 (4%)	11 (4%)	0 (0%)	1.0
Urology	26 (8%)	23 (8%)	3 (30%)	0.04
Orthopedics	29 (9%)	29 (10%)	0 (0%)	0.60
Liver surgery	20 (6%)	19 (6%)	1 (10%)	0.49
Renal transplant	24 (8%)	24 (8%)	0 (0%)	1.0
Other	4 (1%)	4 (1%)	0 (0%)	1.0
Cancer	147 (47.0%)	141 (46.5%)	6 (60%)	0.62
End-stage renal disease	17 (5.4%)	17 (5.6%)	0 (0%)	1.0
**Outcomes**				
ICU length of stay, days	1.1 (+/−3.8)	0.9 (+/−3.4)	5.7 (+/−9.7)	0.15
Hospital length of stay, days	6.8 (+/−10.1)	6.2 (+/−7.8)	25.5 (+/−32.4)	0.09
Myocardial infarction	3 (1%)	3 (1%)	0 (0%)	1.0
Acute kidney injury (RIFLE-risk)	12 (3.8%)	12 (3.9%)	0 (0%)	1.0
**Standard postoperative coagulation studies**				
Prothrombin time, s	32.3 (+/−11.6)	32.3 (+/−11.8)	33.1 (+/−6.2)	0.89
International normalized ratio	1.20 (+/−0.24)	1.20 (+/−0.25)	1.28 (+/−0.11)	0.39
Platelet count	201 (+/−67)	201 (+/−68)	212 (+/−69)	0.63

Data are reported as mean and standard deviation or number and percentage, as appropriate. Obesity = body mass index >30 m/kg^2^. RIFLE, risk, injury, failure, loss, end-stage renal failure; ns = not significant.

There was no significant difference between patients with and without thromboembolic complications with regard to the type of surgery (except for urological surgery) or the presence of preoperative end-stage renal disease (ESRD) or cancer as a cause for surgery (Table 2).

### ROTEM analysis

**Figure 1:**
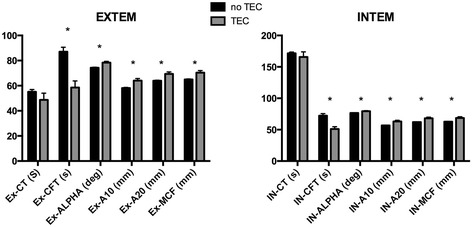
**Preoperative rotational thromboelastometry (ROTEM) parameters using extrinsic rotational thromboelastometry (EXTEM) and intrinsic rotational thromboelastometry (INTEM) activators in patients with and without thromboembolic complications (TEC).** Ex-CT, EXTEM clotting time; IN-CT, INTEM clotting time; CFT, clot formation time; alpha, α angle; A10, amplitude at 10/20 minutes; MCF, maximum clot firmness. *P <0.05.

ROTEM results for patients who did and did not have thromboembolic events are shown in Table 1 and Figure 1. Based on the results of Levene’s test for equality of variance, unequal variance between patients with and without thromboembolic complications was assumed.

Patients with ESRD had smaller α in EXTEM (ESRD versus no ESRD: EXTEM α: 78.1 +/−5.8° versus 74.2 +/−6.9°, *P* <0.05; mean +/−SD). The other ROTEM variables were not significantly different.

When comparing patients with and without thromboembolic complications, clotting time did not differ significantly using any of the three activators (*P* >0.05). For both EXTEM and INTEM, CFT was significantly lower in patients who suffered thromboembolic complications (*P* <0.001 for both). For both EXTEM and INTEM, α, A10 and MCF were higher in patients with than without thromboembolic complications (*P* <0.05 to *P* <0.01). Results using FIBTEM showed similar trends but were not statistically significant.

There was no difference in the incidence of TEC between patients with and without obesity (body mass index (BMI) >30 m/kg^2^ (Table 2)). However, obese patients (n =102) had larger A10 and MCF in EXTEM (A10: 59.8 +/−8.6 versus 57.7 +/−8.7, *P* <0.05; MCF: 66.5 +/−6.8 versus 64.5 +/−7.7, *P* <0.05) and larger MCF in INTEM (64.2 +/−6.2 versus 62.4 +/−7.5, *P* <0.05).

**Figure 2:**
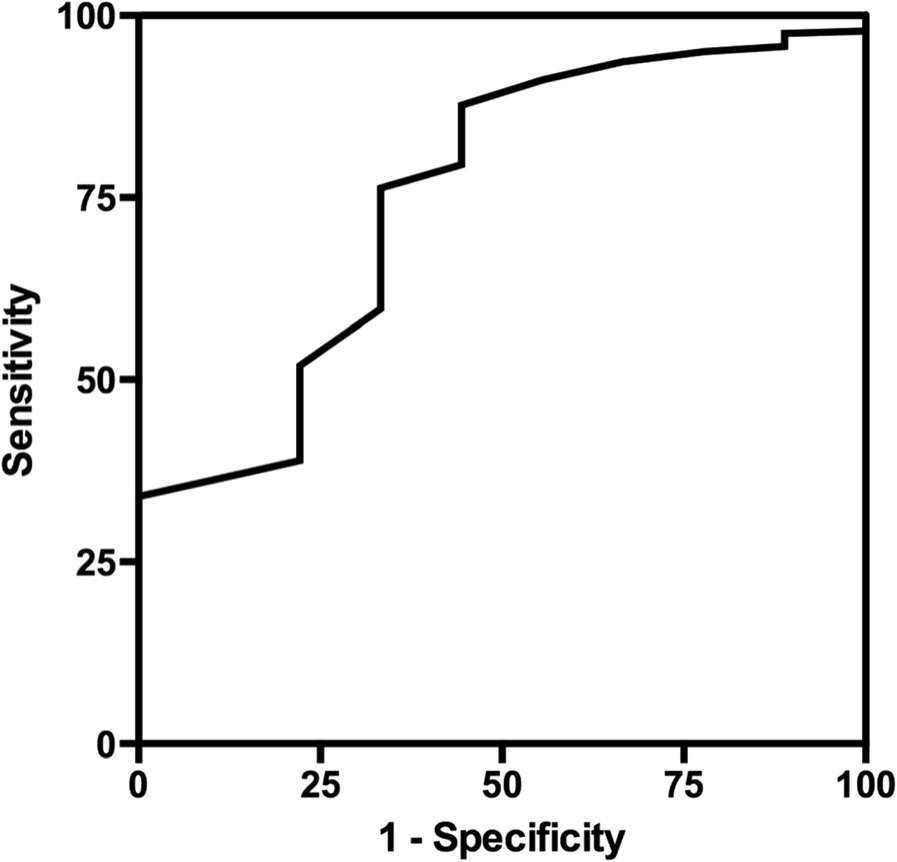
**Receiver operating characteristic curve for amplitude at 10 minutes (A10) using intrinsic rotational thromboelastometry (INTEM) activator.**

ROC curves were plotted for EXTEM and INTEM CFT, α, A10 and MCF. The AUC for these curves are shown in Table 1. INTEM A10 had the largest area under the curve of 0.75. When defining the best cutoff as the point on the ROC curve closest to sensitivity = specificity =1, the best cutoff of INTEM A10 to predict thromboembolic complications was 61.5 mm with a specificity =76.3 (95% CI 70.9% to 81.2%), a sensitivity =66.7 (95% CI 29.9% to 92.51%) and a positive predictive value =8% (95% CI 3.0 to 17.0%) and a negative predictive value =98.0 (95% CI 96.1 to 0.99.7%) and a likelihood ration =2.816. The best cutoff using the largest Youden index (sensitivity + specificity −1) was 64.5 mm with a specificity =87.6 (95% CI 83.2% to 91.2%) and a sensitivity =55.6 (95% CI 21.2% to 86.3%) and positive predictive value =12.5 (95% CI 4.2 to 26.8%) and a negative predictive value =98.4 (95% CI 96.0 to 0.99.6%) and a likelihood ration =4.492. The ROC curve for INTEM A10 is shown in Figure 2.

Hypercoagulability, defined by an MCF above the upper limit of the normal reference values (97.5^th^ percentile of the general population), occurred significantly more often in patients with thromboembolic complications. This held true regardless of whether the definition of hypercoagulability entailed one or two of the three tests as abnormal or only EXTEM as abnormal (odds ratio (OR) =4.73, 5.22, and 4.65, respectively; 95% CI <0.05 for all). These data are summarized in Table 3.

**Table 3:** **Rate of thromboembolic events in patients with hypercoagulability**

**Elevated maximum clot firmness (MCF)**	**Thromboembolic events, % of patients (n)**	***P*** **-value**
**Increased MCF in 2 or 3 of 3 tests (INTEM/EXTEM/FIBTEM)**		
Hypercoagulable	11.5 (3)	
Normal	2.4 (7)	
Odds ratio	5.22	0.01
**Increased MCF in at least 1 of 3 tests (INTEM/EXTEM/FIBTEM)**		
Hypercoagulable	8.6 (5)	
Normal	2.0 (5)	
Odds ratio	4.72	0.009
**Increased MCF in EXTEM**		
Hypercoagulable	9.5 (4)	
Normal	2.2 (6)	
Odds ratio	4.65	0.01

INTEM, intrinsic rotational thromboelastometry; EXTEM, extrinsic rotational thromboelastometry; FIBTEM, fibrinogen rotational thromboelastometry.

## Discussion

Our study found that preoperative ROTEM analysis may be able to identify patients at increased risk of thromboembolic complications after major surgery. Specifically, patients with thromboembolic complications had significantly different ROTEM patterns in EXTEM and INTEM, although not in FIBTEM. A10, A20 and CFT using the INTEM activator were the most reliable predictors of thromboembolic complications, with good predictive power. Patients who developed thromboembolic complication had, on average, decreased CFT and increased α, A10 and MCF compared to patients who did not develop such complications.

These differences have previously been described as signs of hypercoagulability by TEG [7] therefore enhancing the biological plausibility of our results. Although clotting time did not differ significantly between the two groups, CFT, α and MCF together assay the same phase of the clotting cascade: the formation of a stable clot through the interaction of activated platelets and fibrin. The clotting time, in contrast, is the only one of these parameters that measures the first phase of the clotting cascade: the time until fibrin formation. Similarly, standard clotting tests such as PTT and INR reflect the time to fibrin formation only, and, like the clotting time, were not different in patients with and without thromboembolic complications.

The significant indices of hypercoagulability provided by EXTEM and INTEM were not replicated by FIBTEM (for A10/MCF, 0.10 > *P* >0.05). Unlike EXTEM and INTEM, FIBTEM evaluates only the fibrin contribution to clot formation because it incorporates cytochalasin D, a platelet inhibitor. Our findings therefore suggest that tests such as EXTEM and INTEM, which include the platelet contribution to clot formation, are better suited to detect hypercoagulablity. They also enhance our appreciation of the relevance of platelets to the development of hypercoagulability and thromboembolic complications.

A recent meta-analysis by Dai *et al*. [14] highlights several studies suggesting that TEG may be useful in predicting postoperative thromboembolism. However, the authors emphasize the practical limitations of the original, labor-intensive TEG methodology, which may indicate why many past studies included small sample sizes - often 100 patients or fewer, and sometimes as few as ten patients [15]. Larger studies often examined only patients undergoing a specific operation [16,17]. Only one large prospective study has examined the use of TEG in the prediction of thromboembolic events in general surgery [12] and found promising results that require further investigation. In contrast, ROTEM provides a number of advantages that render it much more practicable for routine clinical use at the bedside or in the operating room. These include a stable platform; standardized measuring technique; pathway sub-analysis; rapid, digitized, replicable signatures; and simplicity of operation. At the same time, it is important to understand that although ROTEM is based on the same general technology as TEG, it may produce different results and the devices cannot be assumed to be interchangeable [18].

A few factors should be noted that may contribute to noticeable differences in the incidence of thromboembolic events in this study compared with previous studies using ROTEM or TEG. We chose to include only clinically evident thromboembolic complications; some previous studies incorporated routine deep vein thrombosis screening of all participants. Our goal was to evaluate the clinical applicability of routine ROTEM use, so we excluded subclinical thrombotic events detected by screening tests that might be implemented for study purposes only. We chose not to evaluate thromboembolic risk scores, which identify patients at very high risk of thromboembolic complications and contain many variables that are not readily ascertainable.

Columbia University Medical Center has standardized protocols for thromboembolic prophylaxis, but we neither assessed nor enforced compliance. While it is possible that inadequate compliance may have increased thromboembolic risk, nine out of the ten patients who developed thromboembolic complications did so despite receiving standard thromboembolic prophylaxis. Future studies should be designed to assess not only if preoperative ROTEM is useful to identify high-risk patients, but also whether such patients might benefit from more aggressive or alternative modes of thromboembolic prophylaxis.

Definitions of hypercoagulability are not uniformly established and vary widely in studies using TEG. There are also no clear cutoff values for hypercoagulability when using ROTEM, although an increased MCF is considered the most reliable [10]. Our study confirms that patients with abnormally increased MCF are at increased risk for thromboembolic complications, and may facilitate the development of a standardized ROTEM definition of hypercoagulability. At the same time it must be considered that the reference values for ROTEM have been derived from a healthy volunteer population and not a surgical population. Of note, 2.0 to 2.4% of patients without thromboembolic complications had an MCF higher than the reference value. This is consistent with the definition of the reference value in which 2.5% of the measurements are above the upper limit (97.5^th^ percentile). Nonetheless, the relationship of ROTEM parameters to hypercoagulability remains circumstantial until elucidated by further study. It also needs to be noted that only half of patients with TEC had MCF values that were within the normal limits and only five patients had MCF values that were above the published norm. It will therefore be difficult to detect patients who will develop TECs solely on the basis of ROTEM analysis. Future studies will need to evaluate if inclusion of ROTEM analysis into a broader risk profile with sufficient precision to change clinical management of patients at increased risk. As our study was not designed or powered to establish a change in clinical practice, larger and more focused studies are needed before we can recommend changing thrombembolic prophylaxis solely based on preoperative ROTEM results. The etiology of thromboembolic complications is complex and includes many more factors other than potential inherited or acquired hypercoagulability. We measured ROTEM immediately prior to the surgical procedure to better isolate the effect of hypercoagulability and exclude the many intra- and postoperative factors that contribute to the development of thromboembolic complications and may have affected ROTEM analysis.

Our study did not find that patients undergoing certain classes of surgery or patients with cancer or ESRD had a higher incidence of thromboembolic complications, but it was not designed or powered to detect any possible differences. Interestingly, obese patients had larger MCF in EXTEM and INTEM (and larger A10 in EXTEM). If this is evidence of hypercoagulability associated with obesity [19], this requires further study.

## Conclusions

In summary, we observed that preoperative ROTEM analysis may be able to identify patients undergoing major non-cardiac surgery who are at increased risk for postoperative thromboembolic events. Our study raises a number of pertinent clinical questions. Can defined criteria for hypercoagulability be established by ROTEM? Can ROTEM identify whether high-risk patients could benefit from more aggressive or alternate antithrombotic regimens? How is ROTEM affected by specific inherited or acquired hypercoagulable diseases? And, finally, is routine use of ROTEM cost-effective in helping us to decrease the incidence of postoperative thromboembolic complications?

## Key messages

- Rotational thrombelastography using INTEM and EXTEM prior to non-cardiac surgery is significantly different in patients who develop postoperative thrombembolic complications
- Patients with thrombembolic complications specifically had significantly lower clot formation time (CFT), higher alpha angle (α) and larger maximum clot firmness (MCF)
- INTEM clot firmness at 10 minutes (A10) was the best predictor of thromboembolic complications, with an ROC area under the curve of 0.751
- Rotational thrombelastography may be able to detect patients who are susceptible to postoperative postoperative thrombembolic complications.

## Abbreviations

a: alpha angle
AUC: area under the curve
A10: Clot firmness at 10 minutes
CFT: Clot formation time
CT: computed tomography
ESRD: End-stage renal disease
EXTEM: extrinsic rotational thromboelastometry
FIBTEM: fibrinogen rotational thromboelastometry
ICU LOS: Intensive Care Unit length of stay
INR: international normalized ratio
INTEM: intrinsic rotational thromboelastometry
MA: maximum amplitude
MCF: maximum clot firmness
PTT: partial thromboplastin time
ROC: receiver operating characteristic
ROTEM: rotational thromboelastometry
TEG: thromboelastography
